# An Improved PRoPHET Routing Protocol in Delay Tolerant Network

**DOI:** 10.1155/2015/623090

**Published:** 2015-01-12

**Authors:** Seung Deok Han, Yun Won Chung

**Affiliations:** School of Electronic Engineering, Soongsil University, 511 Sangdo-dong, Dongjak-gu, Seoul 156-743, Republic of Korea

## Abstract

In delay tolerant network (DTN), an end-to-end path is not guaranteed and packets are delivered from a source node to a destination node via store-carry-forward based routing. In DTN, a source node or an intermediate node stores packets in buffer and carries them while it moves around. These packets are forwarded to other nodes based on predefined criteria and finally are delivered to a destination node via multiple hops. In this paper, we improve the dissemination speed of PRoPHET (probability routing protocol using history of encounters and transitivity) protocol by employing epidemic protocol for disseminating message *m*, if forwarding counter and hop counter values are smaller than or equal to the threshold values. The performance of the proposed protocol was analyzed from the aspect of delivery probability, average delay, and overhead ratio. Numerical results show that the proposed protocol can improve the delivery probability, average delay, and overhead ratio of PRoPHET protocol by appropriately selecting the threshold forwarding counter and threshold hop counter values.

## 1. Introduction

In traditional data networks, such as Internet, at least one continuous end-to-end path is guaranteed between a source node and a destination node, and packets are delivered from a source node to a destination node through one of the available paths. In delay tolerant network (DTN), however, an end-to-end path is not guaranteed and packets are delivered from a source node to a destination node via store-carry-forward based routing [1–7]. In DTN, a source node or an intermediate node stores packets in buffer and carries them while it moves around. These packets are forwarded to other nodes based on predefined criteria and finally are delivered to a destination node via multiple hops. A lot of attention has been paid to DTN for possible uses in disconnected network environments, especially for extreme cases such as interplanetary communications [2] and disaster scenarios [7].

One of the representative DTN routing protocols is epidemic routing protocol [8, 9]. As the name implies, a source node forwards a message to all the neighbor nodes whenever it contacts neighbor nodes, like “epidemic.” This simple routing protocol is very powerful even when the buffer size of nodes is sufficient. However, if the buffer size is not sufficient, especially as in mobile nodes, epidemic protocol generates significant message overhead and the performance degrades.

In order to solve the message overhead problem of the epidemic protocol, several schemes have been proposed, such as Spray and Wait protocol [10, 11] and PRoPHET protocol [12, 13]. In these protocols, the total number of message copies present in a network is limited by a certain number or message forwarding is carried out only when a certain condition is met.

The Spray and Wait protocol consists of Spray phase and Wait phase and the total number of message copies present in a network is limited by *L*. In Spray phase, *L* message copies are disseminated to other nodes until there is no node which has more than one message copy. Then, the protocol transitions into Wait phase and the message copy is delivered to a destination node only. In order to enhance the dissemination speed of message copies of basic Spray and Wait protocol, binary Spray and Wait protocol [11] was proposed, where half of stored message copies are distributed to another node. In Spray and Wait protocol, however, message copies are distributed blindly without considering the characteristics of receiving nodes.

In PRoPHET (probability routing protocol using history of encounters and transitivity) protocol, delivery predictability between two nodes is calculated based on contact history between them, where higher delivery predictability implies a higher probability of future contacts between them. In PRoPHET protocol, a message is copied to a contact node only when the delivery predictability to a destination node of the contact node is larger than that of the transmitting node. By doing this, PRoPHET protocol achieves good delivery probability as well as satisfying low overhead.

In PRoPHET protocol, however, we note that the dissemination speed of a message is relatively low, since a message is copied only when a delivery predictability condition is met, and this results in longer average delay and low delivery probability when the buffer size is sufficient. In this paper, we improve the dissemination speed of PRoPHET protocol by employing epidemic protocol for disseminating message *m*, if the conditions for the forwarding counter and the hop counter of message *m* are met. Then, we show that the proposed routing protocol achieves higher delivery probability and lower average delay than PRoPHET protocol.

The remainder of this paper is organized as follows. In Section 2, related works are surveyed. In Section 3, the detailed algorithm of the proposed routing protocol is described. In Section 4, numerical examples are presented using simulation from the aspect of delivery probability, average delay, and overhead. Finally, conclusions and further works are drawn in Section 5.

## 2. Related Works

In related works, works on epidemic and PRoPHET protocols, which constitute the proposed routing protocol, are surveyed in detail.

### 2.1. Epidemic Protocol

Epidemic protocol is basically a flooding-based protocol, and, thus, a node with messages copies them to any other contact nodes if they do not have them already. To do this, two nodes firstly exchange summary vectors which contain the list of messages they have when they contact each other. Then, each node checks the list of messages which it does not have yet and requests the messages from the other node.

If buffer size is infinite, epidemic routing protocol can achieve optimal delivery probability and average delay. Since buffer size is finite and epidemic protocol generates significant copies of a message, enhanced schemes to manage limited buffer and battery energy have been proposed. As an example, in an energy efficient *n*-epidemic routing protocol [14], a node copies messages to other nodes only when the number of neighbor contact nodes reaches a predefined threshold value, that is, *n*, in order to save energy.

In [15], the authors studied the performance of four categories of epidemic routing protocol in detail, that is, P-Q epidemic, epidemic with time-to-live (TTL), epidemic with encounter counter (EC), and epidemic with immunity table using trace-file and random waypoint mobility models. Then the authors proposed three enhanced schemes such as dynamic TTL, EC + TTL, and cumulative immunity and showed that the enhanced schemes can improve delivery probability and buffer occupancy level can be reduced significantly for cumulative immunity scheme.

### 2.2. PRoPHET Protocol

PRoPHET protocol uses nonrandom mobility and contact patterns in real application scenarios to copy messages to other nodes in order to improve the routing performance [13]. That is, the PRoPHET protocol is based on the fact that if a node has visited a location or contacted with a node frequently, the probability of visiting the location and contacting the node is higher. To achieve this, “delivery predictability” is defined at a node for every other contacted node. The delivery predictability of node A to node B is denoted by *P*_A,B_ and the range of delivery predictability value is defined as 0 ≤ *P*_A,B_ ≤ 1. If node A with a message to a destination node D contacts with node B, node A and node B exchange summary vectors and delivery predictability. Then, node A compares *P*_A,D_ and *P*_B,D_. If *P*_B,D_ > *P*_A,D_, the message to destination node D is copied to node B. Otherwise, the message is not copied to node B.

In PRoPHET+ [16], deliverability is defined as a weighted sum of buffer size, battery power, location, popularity, and the delivery predictability. If a node meets another node, it queries deliverability value of another node. Then, if the deliverability value of another node is higher than a predefined threshold value, a source node sends a message to another node. If there are multiple nodes which are in simultaneous contact with the source node, the message is sent to the node with the highest deliverability value. In PRoPHET+, it was shown that the proposed PRoPHET+ performs well from the aspect of delivery probability and average delay, by appropriately choosing the weight factor for buffer size, battery power, location, popularity, and the delivery predictability.

In [17], a policy of history of message's movement was newly considered and a new probabilistic routing protocol based on history of message was proposed. In the proposed protocol, message's hop count as well as delivery predictability is considered to determine next hop node, where the history of message's traversed path is defined as a sequence of contacted nodes.

In distance-based PRoPHET [18], distance between two nodes was additionally used to compute delivery predictability. That is, each node having a message checks distance from neighboring nodes and chooses a node located in a smaller distance as a forwarder since a node can have higher transmission rate to a nearer node and, thus, can increase the delivery probability and decrease delivery delay. In [19], the authors extended the results in [18] by considering community mobility model, in addition to random waypoint mobility model in [18].

## 3. An Improved PRoPHET Routing Protocol

The proposed improved PRoPHET routing protocol is a hybrid of epidemic protocol and PRoPHET protocol. The main idea of the proposed protocol is to accelerate the dissemination of messages in the early phase of message delivery, by employing epidemic protocol. On the other hand, the proposed protocol restricts dissemination in later phase since it only copies messages to other nodes only when a delivery predictability condition is met.

Before going into the detailed operation, new notations are defined as follows.

- *n*_*m*_ is forwarding counter of message *m* at the current node, which is defined as the total number of messages copied to other nodes for the message *m* along the forwarding path from a source node to the current node. At the generation of message *m*, the value of *n*_*m*_ is initialized at 0. If the current node is a source node, *n*_*m*_ for a message *m* means that the source node forwarded *n*_*m*_ copies to other nodes. If the current node is not a source node, the initial value of *n*_*m*_ at the current node was set to the current *n*_*m*_ value of the previous transmitting node and *n*_*m*_ was increased by one for each message copy to another node from the current node.
- *N*_*m*_ is threshold forwarding counter of message *m*.
- *h*_*m*_ is hop counter of message *m* at the current node, which is defined as the total number of hops that a message *m* has traversed along the forwarding path from a source node to the current node. If the current node is a source node, *h*_*m*_ for a message *m* is initialized at 0.
- *H*_*m*_ is threshold hop counter of message *m*.

Algorithm 1 shows the detailed algorithm of the proposed protocol. At the generation of message *m*, both the values of *n*_*m*_ and *h*_*m*_ are initialized as 0. When node *i* contacts node *j*, they exchange summary vectors which contain message list stored in each node. Then node *i* decides candidate message set *M* to transmit to node *j*. After selecting a message *m* ∈ *M*, node *i* checks the relationship between *n*_*m*_ and *N*_*m*_. If *n*_*m*_ > *N*_*m*_, PRoPHET routing protocol is used to deliver the message *m*. Otherwise, node *i* checks again the relationship between *h*_*m*_ and *H*_*m*_. If *h*_*m*_ > *H*_*m*_, PRoPHET routing protocol is used to deliver the message *m*, too. Then, message *m* is delivered using PRoPHET routing protocol and if it is delivered to another node, *n*_*m*_ of the receiving node is initialized at the current value of *n*_*m*_ of the transmitting node and *h*_*m*_ of the receiving node is increased by one (*h*_*m*_++). Otherwise, that is to say, if *n*_*m*_ ≤ *N*_*m*_ and *h*_*m*_ ≤ *H*_*m*_, node *i* uses epidemic routing protocol to deliver the message *m*. Then *n*_*m*_ of the transmitting node is increased by one (*n*_*m*_++) and the increased *n*_*m*_ and *h*_*m*_++ information is delivered to the receiving node too. The loop of delivering a message in *M* repeats until all the messages are delivered based on either epidemic or PRoPHET routing protocol.

**Figure 1:**
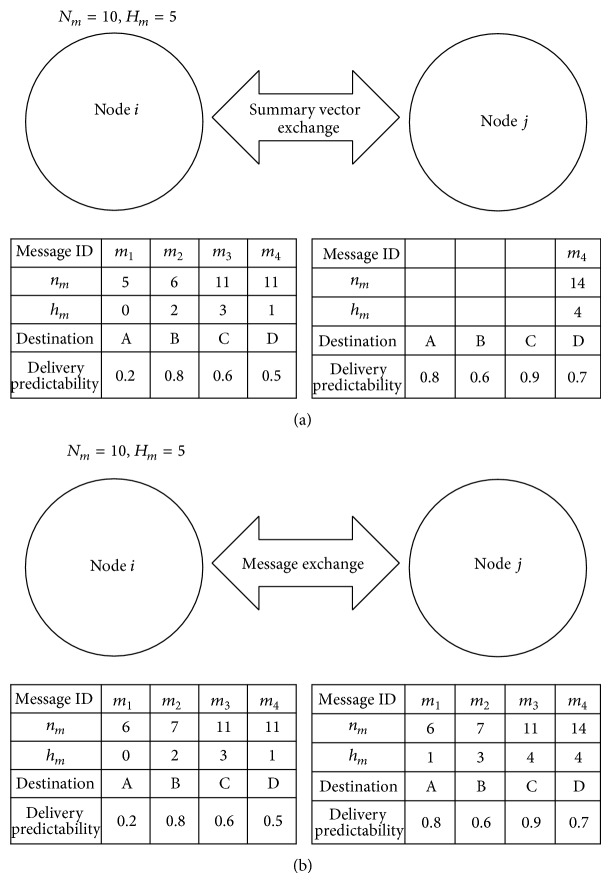
Example scenario of the proposed protocol ((a) summary vector exchange, (b) message exchange).

Figure 1 shows an example scenario of the proposed protocol in DTN environment, where *H*_*m*_ = 5 and *N*_*m*_ = 10. Suppose that node *i* has four messages, that is, *m*_1_, *m*_2_, *m*_3_, and *m*_4_, and node *j* has one message *m*_4_. If they are within the contact of each other, they exchange summary vectors which contain message list stored in each node and delivery predictability information. In the considered scenario in Figure 1, node *i* determines that messages *m*_1_, *m*_2_, and *m*_3_ should be delivered to node *j*. Then, node *i* delivers *m*_1_ and *m*_2_ using epidemic protocol since *m*_*i*_ ≤ *M*_*i*_ and *h*_*i*_ ≤ *H*_*i*_ for *i* = 1,2, and the values of *n*_1_ and *n*_2_ are increased by one in both nodes *i* and *j*. Also, the values of *h*_1_ and *h*_2_ in node *j* are increased by one from those in node *i*. Node *i* delivers *m*_3_ using PRoPHET protocol since *n*_3_ > *N*_3_. Since delivery predictability to destination node C in node *j* is larger than that in node *i*, the message *m*_3_ is delivered to node *j* using PRoPHET protocol and *h*_3_ in node *j* is increased from that in node *i*.

## 4. Numerical Examples

In this paper, we carried out simulation for the proposed protocol using the opportunistic network environment (ONE) simulator developed by Helsinki University [20, 21]. In numerical examples, we analyzed the performance of the proposed protocol, from the aspect of delivery probability, average delay, and overhead ratio, for varying the values of threshold forwarding counter and threshold hop counter. The delivery probability is defined as the number of successfully delivered messages divided by the number of created messages. The average delay is the average value of delay for successfully delivered messages. The overhead ratio is defined by (NR − ND)/ND, where NR is the number of relayed messages and ND is the number of successfully delivered messages. The parameter values are assumed, as in Table 1.

### 4.1. The Effect of Threshold Forwarding Counter *N*_*m*_

**Figure 2:**
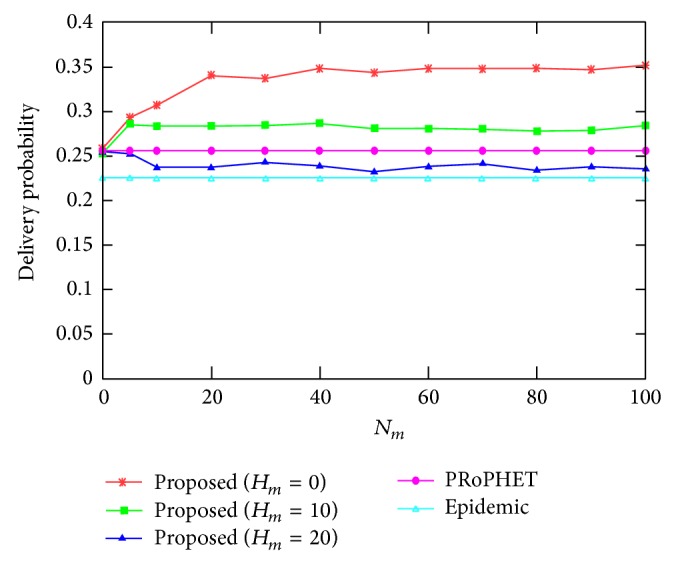
Delivery probability for varying the value of *N*_*m*_.

Figure 2 shows the delivery probability for varying the values of *N*_*m*_, where *H*_*m*_ = 0,10, and 20. The delivery probability of the proposed protocol when *H*_*m*_ = 0 and 10 is higher than that of PRoPHET and epidemic protocols, which are constant since they do not depend on the values of *N*_*m*_, for most values of *N*_*m*_. This is because the proposed protocol employs epidemic protocol to spread the message copies quickly until the forwarding counter value reaches threshold forwarding counter value and employs PRoPHET protocol to deliver message copies to better nodes with higher delivery predictability to destination nodes after the forwarding counter value reaches threshold forwarding counter value. However, the delivery probability of the proposed protocol when *H*_*m*_ = 20 is lower than that of PRoPHET protocol, since too many message copies result in message drop in buffer and, thus, reduce delivery probability compared to PRoPHET protocol. The delivery probability of the proposed protocol when *H*_*m*_ = 20 is, however, still higher than that of epidemic protocol, since the number of message copies is still smaller than that of epidemic protocol. The delivery probability of the proposed protocol saturates as the value of *N*_*m*_ becomes large since the effect of increasing the value of *N*_*m*_ for large values of *N*_*m*_ is negligible.

**Figure 3:**
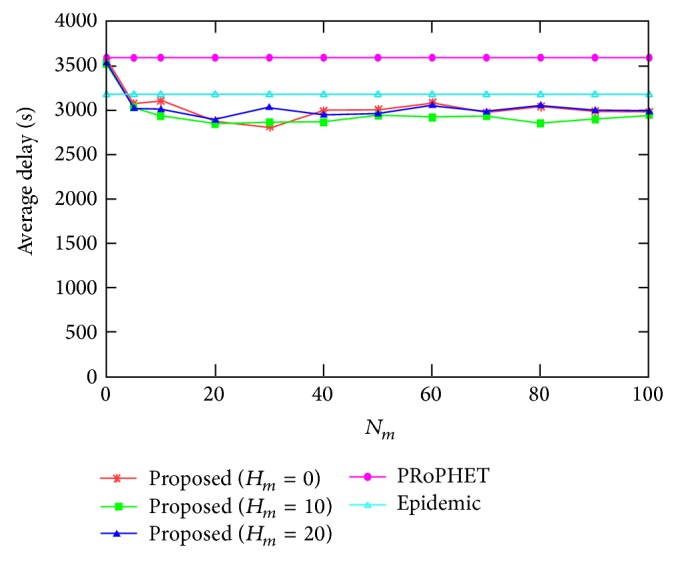
Average delay for varying the value of *N*_*m*_.

Figure 3 shows the average delay for varying the values of *N*_*m*_, where *H*_*m*_ = 0,10, and 20. The average delay of the proposed protocol is smaller than that of PRoPHET and epidemic protocols for most values of *N*_*m*_, since the proposed protocol reduces the average delay of PRoPHET protocol by employing epidemic protocol in source node, but the packet is delivered more quickly than epidemic protocol since the proposed protocol uses delivery predictability for better delivery and uses more message copies for fast delivery compared to PRoPHET protocol. Similar to Figure 2, the effect of *N*_*m*_ on the average delay becomes negligible as the value of *N*_*m*_ becomes large.

**Figure 4:**
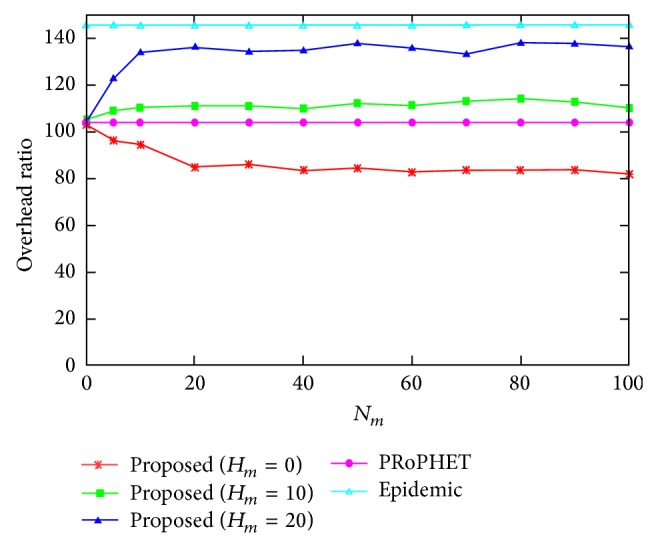
Overhead ratio for varying the value of *N*_*m*_.

Figure 4 shows the overhead ratio for varying the values of *N*_*m*_, where *H*_*m*_ = 0. The overhead ratio of the proposed protocol when *H*_*m*_ = 0 is smaller than that of both PRoPHET and epidemic protocols, since the effect of the increased number of delivered messages of the proposed protocol is higher than that of the increased number of relayed messages of the proposed protocol for *H*_*m*_ = 0. However, the overhead ratio of the proposed protocol when *H*_*m*_ = 10 and 20 is higher than that of PRoPHET protocol since the effect of the increased number of relayed messages of the proposed protocol is higher than that of the increased number of delivered messages of the proposed protocol. Similar to Figures 2 and 3, the effect of *N*_*m*_ on the overhead ratio becomes negligible as the value of *N*_*m*_ becomes large.

### 4.2. The Effect of Threshold Hop Counter *H*_*m*_

**Figure 5:**
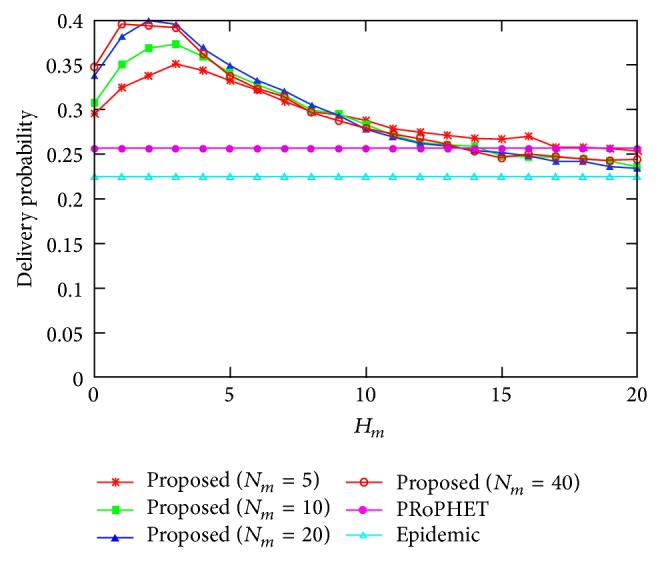
Delivery probability for varying the value of *H*_*m*_.

Figure 5 shows the delivery probability for varying the values of *H*_*m*_, where *N*_*m*_ = 5,10,20, and 40. The delivery probability of the proposed protocol is higher than that of epidemic protocol always. Also, the delivery probability of the proposed protocol is higher than that of PRoPHET protocol for most values of *H*_*m*_; that is, *H*_*m*_ ≤ 15, and it is slightly lower than that of PRoPHET protocol for large values of *H*_*m*_, since too many message copies result in message drop in buffer and thus, reduces delivery probability of the proposed protocol. The delivery probability of the proposed protocol increases as *H*_*m*_ increases from *H*_*m*_ = 0 and decreases as *H*_*m*_ increases from *H*_*m*_ = 2 or 3 in the considered parameter setting. This is because increasing the spreading of message copies from *H*_*m*_ = 0 to *H*_*m*_ = 2 has a positive effect on increasing delivery probability due to higher message copies but the increasing the spreading of message copies too high, that is, from *H*_*m*_ = 2 or 3, results in higher buffer occupancy and this results in message drop. The delivery probability of the proposed scheme when the values of *H*_*m*_ are small increases as *N*_*m*_ increases but it is saturated for large values of *H*_*m*_.

**Figure 6:**
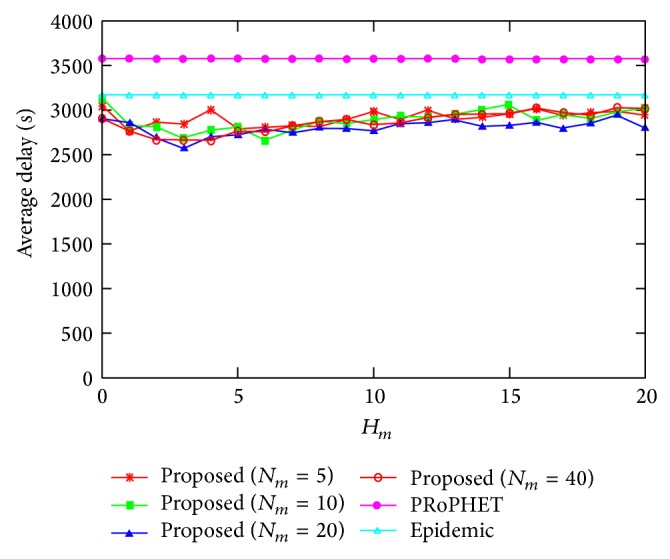
Average delay for varying the value of *H*_*m*_.

Figure 6 shows the average delay for varying the values of *H*_*m*_, where *N*_*m*_ = 5,10,20, and 40. Similar to Figure 3, the average delay of the proposed protocol is smaller than that of PRoPHET and epidemic protocols for most values of *H*_*m*_, based on a similar rationale as in Figure 3. From Figure 6, it can be shown that the effect of different values of *N*_*m*_ on the average is not significant.

**Figure 7:**
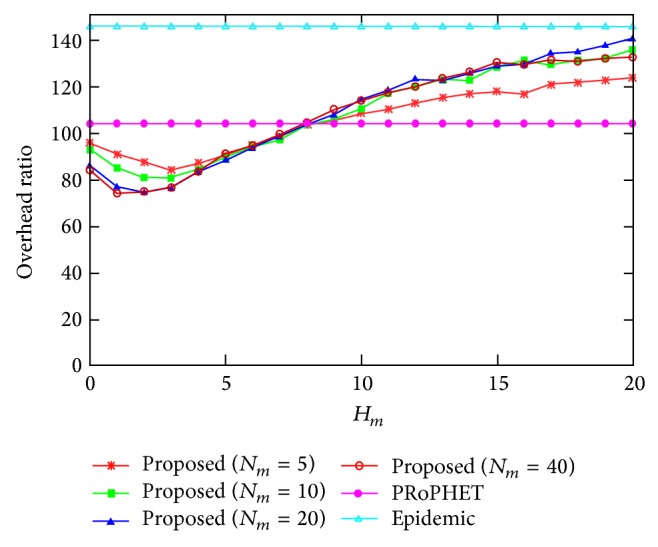
Overhead ratio for varying the value of *H*_*m*_.

Figure 7 shows the overhead ratio for varying the values of *H*_*m*_, where *N*_*m*_ = 5,10,20, and 40. The overhead ratio of the proposed protocol is always smaller than that of epidemic protocol, since the proposed protocol generates smaller message copies. The overhead ratio of the proposed protocol is smaller than that of PRoPHET protocol for small values of *H*_*m*_; that is, *H*_*m*_ ≤ 8, since the effect of the increased number of delivered messages of the proposed protocol is higher than that of the increased number of relayed messages of the proposed protocol for *N*_*m*_ = 5,10,20, and 40. However, the overhead ratio of the proposed protocol is higher than that of PRoPHET for large values of *H*_*m*_, since the effect of the increased number of relayed messages of the proposed protocol is higher than that of the increased number of delivered messages of the proposed protocol with *N*_*m*_ = 5,10,20, and 40.

## 5. Conclusions and Further Works

In this paper, we improved the dissemination speed of PRoPHET protocol by employing epidemic protocol if forwarding counter and hop counter values are smaller than or equal to the threshold values. Then, the performance of the proposed protocol was analyzed from the aspect of delivery probability, average delay, and overhead ratio for varying the values of threshold forwarding counter and threshold hop counter using ONE simulator. Numerical results show that the proposed protocol can improve the delivery probability, average delay, and overhead ratio of PRoPHET protocol, by appropriately selecting the threshold forwarding counter and threshold hop counter values. As further works, we will propose an adaptive selection of threshold forwarding counter and threshold hop counter based on measured network parameter values to maintain good performance for varying network environments always.

## Figures and Tables

**Algorithm 1:**
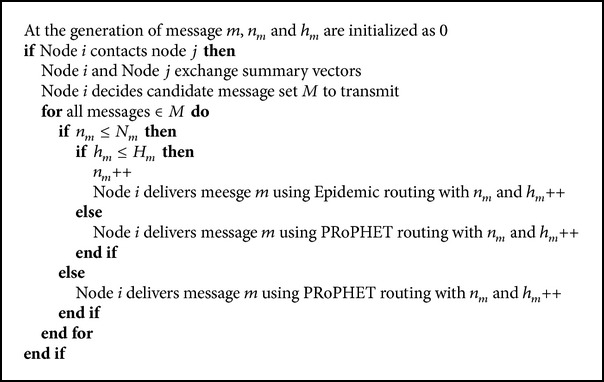
Detailed algorithm of the proposed scheme.

**Table 1:** Parameter values.

Parameter	Value
Area size (m^2^)	4,500 × 3,400

Simulation times (s)	21,600

Transmission range (m)	10

Transmission rate (kbps)	250

Movement model	Tram: MapRouteMovement
	Car, pedestrian: ShortestPathMapBasedMovement

Speed of nodes (m/s)	Tram: *U*[5,14]
	Car: *U*[2.7 : 13.9]
	Pedestrian: *U*[0.5 : 1.5]

Buffer size (bytes)	Car, pedestrian: 5 M
	Tram: 50 M

Message generation intervals (s)	*U*[25,35]

Message size (kbytes)	*U*[500, 1,000]

Number of nodes	Tram: 6
	Car: 40
	Pedestrian: 80
